# Outcome of Chair Aerobics & Pranayama on Anxiety and Exercise Tolerance in Coronary Artery Bypass Grafting Patients: Study Protocol of a Randomized Clinical Trial

**DOI:** 10.29337/ijsp.166

**Published:** 2021-10-21

**Authors:** Abeeshna Ashok, K. U. Dhanesh Kumar, Mundayat Gopalakrishnan

**Affiliations:** 1 Nitte Institute of Physiotherapy, NITTE (Deemed to be University), Mangalore, Karnataka, India; 2 Department of Cardiothoracic and vascular surgery, K.S. Hegde Medical Academy, NITTE (Deemed to be University), Mangalore, Karnataka, India

**Keywords:** Cardiac rehabilitation, Coronary Artery Bypass Grafting, Anxiety, Pranayama, Chair aerobics

## Abstract

**Objectives:**

The current Indian scenario follows the western cardiac rehabilitation protocol; hence the primary aim of the study is to develop a cardiac rehabilitation phase 1 protocol for the Indian scenario. The protocol will be used in the study as standard rehabilitation protocol for the intervention groups. The literature suggests the use of Pranayama and chair aerobics to improve the anxiety in CABG patients. This study also aims to provide the answer for the effect of Pranayama and chair aerobics on anxiety and exercise tolerance in CABG patients. And also try to find out which among the two intervention is superior among one another.

**Methods:**

The cardiac rehabilitation protocol will be validated by experts in the field and applied in the patients and the results will be analysed. Then the protocol will be used as the standard rehabilitation protocol in both the groups. 100 patients will be randomised and allocated into 2 groups. Group 1 will receive Nadi Sodhana Chair aerobics for 15 minutes along with phase I cardiac rehabilitation. The group 2 will receive Chair aerobics for 15 minutes along with phase I cardiac rehabilitation. The outcome measures will be taken before the surgery and on the post-operative day 7. The primary outcome measures are Hospital anxiety and depression scale (HADS) and Heart rate and the secondary outcome measure is 6-minute walk test. The intention to treat analysis will be done after the data collection.

**Results:**

The data will be analysed using unpaired t test, p value <0.05 will be considered significant.

**Conclusion:**

The result will give a new insight into the field of cardiac surgery, where the effect of pranayama and chair aerobics on anxiety and functional outcome will be proved.

**CTRI registration:**

This trial is prospectively registered in CTRI, the registration number of the trial is **CTRI/2021/09/037008**.

**Highlights:**

**What is already known about this subject?** The effect of Pranayama and chair aerobics on various components like pain, peak expiratory flow after CABG is proven in different studies. The phase 1 cardiac rehabilitation is practiced and adopted from western protocol.

**What does this study add?** The study will give a new insight into the field of cardiac rehabilitation. Definite phase I cardiac rehabilitation protocol for Indian population is not exist in the literature. The Indian set up is using the western protocol, which is not suitable for the Indian population hence could not achieve the expected outcome on discharge. We believe that this study will provide a definite phase I cardiac rehabilitation protocol for the Indian population. This can be followed in the community. Also, this study aims to explore the unexplored area of anxiety after CABG. Where the effect of the Pranayama and chair aerobics will be identified. And also give idea about which treatment technique is superior, and feasible for the patients.

**How might this impact on clinical practice?** The study will provide a new phase I cardiac rehabilitation protocol for the Indian population. The protocol can be practiced in the Indian scenario. This will help to improve the exercise tolerance of the patients after the surgery. The study will recommend the feasible and effective technique for relieving the anxiety and improving the exercise tolerance in CABG patients. This can be implemented as a best practice in reducing anxiety after CABG.

## Introduction

Coronary Artery Disease (CAD) is an epidemic in India which is the result of epidemiological transition, contributed by industrialisation, urbanisation, and lifestyle changes [1]. The inner wall of the artery becomes narrowed and hardened in CAD. Global Burden of Disease (GBD) report 422.7 million people in the world are having CAD, and prevalence is more in Indian population compared to other countries in the same region. World Health Organisation (WHO) and GBD studies reveal that years of life lost (YLLS) and disability adjusted life years (DALYS) raised from 1 to 9–10% in the urban India and <1% to 4–6% [2]. By the end of 20^th^ century CAD contributed 50% of death in developed countries and 25% death in developing countries [3]. The plaque deposit in the artery causes narrowing of the lumen of the artery. Blood supply to the myocardial muscle tissue is compromised in the distal part of occlusion. The heart muscles become unable to contract and the blood supply from the heart is compromised. This leads to unstable angina, myocardial infarction, and sudden death [4]. The risk factors leading to the same is classified as modifiable and non-modifiable. Age, gender, ethnicity, and family history are the factors which cannot be modified. The modifiable factors are hypertension, hyperlipidaemia, diabetes mellitus, obesity, smoking, unhealthy diet, and sedentary lifestyle [5]. Risk factor modification is the mainstay of treatment in the early stages of disease and beneficial as preventive measure [6]. In the initial phases the disease requires conservative medical management [7]. Coronary angiogram is the most widely used diagnostic tool in detecting the severity of stenosis. A stenosed artery above 70% should be repaired surgically, known as coronary artery bypass grafting (CABG)[8]. It is the common surgical technique involving diverting the blood supply to the occluded vessels and replenishing the blood supply of the myocardium. The life expectancy is very high. There are many complications associated with the surgery which may affect the prognosis and quality of life in patients, which accounts 25%. There are physical and mental complications. The physical complications include myocardial infarction, stroke, and even kidney failure and the mental complications include such as mood disorders, fatigue, weakness, stress, anxiety, and depression [9]. Depression and anxiety are the most important factors which badly affect the prognosis of the patient. International Classification for Diseases-tenth edition (ICD-10) depression is defined as the by “low mood and/or anhedonia (loss of interest in activities that once were pleasurable) that lasts for two weeks or more and is accompanied by significant functional impairment and somatic complaints of disturbed sleep, fatigue, body aches, digestive or sexual problems, and negative thoughts”. Defines anxiety as a feeling of apprehension and unease. It has three components. Somatic symptoms include digital tremors, palpitations, and sweaty palms. The physiological component mentions tachycardia, hyperventilation, muscular tension, and an irritable bladder. The cognitive component is the undue fear of something untoward happening. The rate of depression and anxiety is increasing in Indian population due to the socioeconomic factors, fear of death etc. [10] Depression has a long-term effect on CABG patients which accounts for 25% of the population. Anxiety is associated with increased sympathetic activity and reduced parasympathetic activity [11]. Anxiety is a complex psychological disorder which is connected to both physiological and psychological response [12]. This in turn reduces the exercise tolerance. Henceforth alleviation of symptoms of anxiety and depression is utmost important in post CABG patients. And this should be cost effective for the patient, because of the economic concerns which leads to these symptoms. Cardiac rehabilitation is the routine care provided after CABG. It is a multi-disciplinary individualised approach. Various models are available for cardiac rehabilitation, and diverse protocols are being practiced in various countries [13]. Cardiac rehabilitation has an impact of anxiety, which alleviates anxiety [14]. According to ACSM the preferred intensity of phase I cardiac rehabilitation is, Heart rate 120 beats per minute [15]. The inpatient rehabilitation is 3–4 weeks in Austria, and central and eastern Europe [16]. The cardiac rehabilitation protocol in the existing literature is more towards the western culture. The definite protocol for Indian population is adopted from various western countries. So, developing a protocol for the Indian set up is obligatory. Hence the primary objective of the study is to develop the phase 1 cardiac rehabilitation protocol for Indian population. And the same protocol will be validated by various experts in the field and apply this in the patient population and measure the outcome using 6 Minute walk. The same protocol can be used in the current study as the standard treatment protocol. To lessen the anxiety literature suggests the use of pranayama, yogic intervention, includes prolongation and control of breath. Yogic intervention may improve the anxiety among patients undergoing CABG, but lacks literature supporting the similar [17]. Practice of pranayama has an effect on the autonomic system function, and thereby controls anxiety. Pranayama means the control of ‘Prana’ in Indian philosophy, refers to all forms of energy in the universe. Slow and deep breathing technique, “Nadi-shodhana Pranayama” increases parasympathetic activity. But there is a deficiency of literature to prove the effect of “Nadi-shodhana Pranayama” on anxiety in CABG patients [18]. Improving the aerobic capacity can increase peak oxygen consumption and reduce anxiety. Chair aerobics is a form of low intensity activity which can be given for post CABG patients [19]. The literature is deficient to provide evidence of chair aerobics in improving the exercise tolerance and anxiety in patients after CABG. Extensive literature is required prove the effect of “Nadi-shodhana Pranayama” pranayama and chair aerobics in reducing the anxiety and improving the exercise tolerance in the field of CABG.

## Materials and Methods

The trial design is Randomized clinical Trial, outcome assessor blinded double arm trial. The participants will be recruited from department of cardio-thoracic and vascular surgery, Justice K S Hegde charitable hospital.

## Selection Criteria

### Inclusion criteria

- CABG patients
- Age: 30–80 years
- HADS anxiety cut off score 7
- Both genders

### Exclusion criteria

- Patients with multiple procedures (Eg: CABG+ valve replacement)
- LVEF < 40%
- Patients who receive anxiolytics, sedatives, or hypnotics
- Cardiac Failure
- Heart Transplantation
- Essential Hypertension
- Diabetic Autonomic Neuropathy
- Neurological Disorders
- Patients on mechanical ventilator

## Procedure

Interventions: Ethical approval is obtained for the study. Verbal advertisement will be given to the patients of JUSTICE K S HEGDE CHARITABLE HOSPITAL who is undergoing cardiac surgery (CABG) and willing to participate in the study.

Written permission to conduct this study is obtained from the Head of the cardio-thoracic and vascular surgery Department. Informed consent will be obtained from patients. Following the completion of baseline data collection, patients will be allocated into two groups based on computer generated randomization and sequentially numbered, opaque, sealed envelopes (SNOSE).

The study has 2 phases. In first phase the phase 1 cardiac rehabilitation will be validated by experts and then will be applied to patients and obtain the results. In the second phase, the proposed rehabilitation protocol will be applied to the patients in both the groups.

1. Baseline data will be obtained prior to intervention (including heart rate, respiratory rate, pulse rate, BMI, RPE, 6-minute walk test, HADS, history regarding any medical treatment and other health related conditions).
2. Prior to intervention (on pre-operative day) outcome scores will be taken.
3. Patients will be randomized into 2 groups based on the SNOSE method.
4. **Group 1 will receive**
**Yoga therapy (Pranayama)**
  The yoga will be delivered by a trained personal. The Nadisodhana pranayama will be delivered once in a day. The vitals will be monitored throughout the procedure and the position will be according to the cardiac rehabilitation.
**Nadi Sodhana Pranayama**
  - The individual will be made in Sukhasana (comfortable posture). Patient will be instructed to open the right hand and bend the index finger bent index and middle fingers against the palm. Close the right nostril using the thumb, while the fourth and fifth fingers will used for the left nostril. The patient will be then instructed to place the right thumb against the ala at the end of the nostril to close it and similarly pressed the fourth and fifth fingertips against the left nostril. The procedure will be continued for 15 minutes [20].
5. **GROUP 2 will receive**
**Chair aerobics**
**POD 3–4**
  1. Alternate heel digs with bilateral biceps curl – 8 repetitions
  2. V- step with bilateral hammer curls – 8 repetitions
  3. Lateral step touch with bilateral biceps curl – 8 repetitions
  4. Knee lift – 8 repetitions
**POD 5–7**
  1. Alternate heel digs with bilateral arm curl – 16 repetitions
  2. V- Step with bilateral hammer curls – 16 repetitions
  3. Lateral step touch with bilateral arm curl – 16 repetitions
  4. Knee lift – 16 repetitions
  The intensity of the exercises is low and as tolerated by the subject based on RPE Borg’s scale. The total time duration will be 15 minutes.
  And will receive cardiac rehabilitation phase 1 protocol.
6. All the outcome measures such as HADS, 6MWT and HR, will be taken on preoperative day and on POD 7.
7. Statistical analysis will be done after completing the sample collection. Intention to treat analysis will be done.

## Sample Size

Based on the mother article, standard Deviation in the intervention group is 6.10 beats per minute [19]. Mean difference for effect size is 0.6483, alpha error 5%, and power 90%. The required samples per group is 50. The total sample size is 100.

## Results

Intention to treat analysis will be done after completing the sample collection.

Data will be analysed using unpaired t test. p < 0.05 will be considered significant.

## Limitations of the study

The study aims to develop a phase 1 cardiac rehabilitation protocol, the phase 2, 3 and 4 is beyond the scope of the study. The study duration is phase 1 cardiac rehabilitation the prolonged effect of the Pranayama and chair aerobics is beyond the scope of the study.

## Discussion

Price KJ et al put forward a question that is there any international consensus for cardiac rehabilitation? They found and suggested that a new protocol guided with aerobic exercise and resistance training should be formulated. This study is the base for the first stage of our study which aims to formulate a phase 1 cardiac rehabilitation. [13] we are aiming at this primary objective in the first phase of the study. Babu A et al in their non-randomized experimental study, prospectively enrolled patients. They received phase-1, exercise-based, protocol-guided CR. They suggested that protocol-guided, phase-1 CR produces a much faster return of heart rate and blood pressure to baseline following the 6MWT, without creating a great rise in the RPE throughout the 6MWT, which suggests a training benefit among these patients. They recommended larger studies to validate these results. We aim to implement the newly developed protocol, validate the same and compare the results of the study with the study results conducted by Babu et al. [15] Shah MR et al in their study evaluated the effect of pranayama on pain and length of hospital stay, conventional exercises along with pranayama were given to the patients. The study results found that there is a reduction in the post-operative depressive function [18] In the second phase of the study we compare the effect of Nadi sodhana pranayama and chair aerobics in CABG patients. They also suggested future studies with larger sample size. Our study aims to find out the effect of Nadishodhana pranayama on anxiety and exercise tolerance with a larger sample size, so that the effect can be validated. Thapa S et al in their single centre prospective study, demonstrated the effect of chair aerobics on vital parameters and exercise tolerance. They suggested the intervention in improving the functional capacity of the patients after CABG who receives cardiac rehabilitation [19]. In this study we also aim to prove the effect of chair aerobics on anxiety and functional capacity in CABG population. This will give a new insight into the field of anxiety after CABG. Manikumar et al included thirty patients with CABG in their experimental pre-test and post-test design. They established that nadisodhana pranayama reduced the intensity of the pain and improve chest expansion and peak expiratory flow on post-operative day 6 [20]. Our study aims to explore the unexplored area of anxiety in CABG patient. Studies have also explored the area of air way inflammation, air way collapse, chest expansion, and air way oxygenation along with pulmonary function tests by using nadisodhana pranayama (Karunakara Padhy et al). [21] Our study aims to find the ANS response by using heart rate of the patient. Chandrababu R et al in their study provided pranayama in cardiac surgery patients and found that the technique is improving the anxiety. However, they recommended high quality randomized controlled trial with a larger sample size.[22] We hope to provide a high quality research for the same.
